# Bovine Trypanosomosis: Seasonal Prevalence and Vector Density in Dara District, Sidama Region, Ethiopia

**DOI:** 10.1002/vms3.70130

**Published:** 2024-11-27

**Authors:** Tafese Jiso, Gizachew Hailegebreal, Dessie Shiferaw

**Affiliations:** ^1^ Dara District Livestock Office Sidama Regional State Dara Ethiopia; ^2^ Faculty of Veterinary Medicine Hawassa University Hawassa Ethiopia

**Keywords:** cattle, dara district, glossina, PCV, season, trypanosomosis

## Abstract

In sub‐Saharan Africa, animal trypanosomosis is a wasting disease that reduces livestock's health and productivity. A recurrent cross‐sectional investigation was carried out in the Dara district of the Sidama region in dry and wet seasons to estimate the apparent density of *Glossina* spp. and the seasonal prevalence of bovine trypanosomosis. Study animals were selected by systematic random sampling, and a total of 388 blood samples were analysed using Giemsa‐stained thin blood smear and Buffy coat methods in both the wet and dry seasons. To conduct a study on tsetse and biting flies, 80 odour‐baited NGU traps were placed near the grazing and watering locations. The overall prevalence of trypanosomosis was 4.4% (95% CI = 2.7–7.0), of which 1.5% and 7.2% accounted for dry and wet seasons, respectively. The prevalence of *Trpanosoma congolense*, *Trypanosoma vivax* and mixed infection (*T*. *congolense* and *T*. *vivax*) was 1.6% (95% CI = 0.3–2.8), 1.3% (95% CI = 0.2–2.4) and 1.6% (95% CI = 0.3–2.8), respectively. The prevalence of trypanosomosis was significantly higher in the wet season than dry season (OR = 5, *p* < 0.05) and in black coat colour animals than in the other coat colour animals (OR = 6.6, *p* < 0.05). The mean PCV of parasitaemic animals (21.2 ± 0.5) was significantly lower than that of aparasitaemic animals (27.7 ± 0.2). A total of 931 flies were caught, of which 154 (16%) were tsetse flies, while 148 (16%) were *Tabanids* and 136 (15%) were *Stomoxys*. In the research area, *Glossina pallidipes* was the only spp. identified. The overall mean apparent density of *G. pallidipes* was 0.96 F/T/D. *G. pallidipes* were caught in comparatively greater numbers during the wet season than during the dry season. Overall, the results of this study demonstrated that the region's cattle production is threatened by *Glossina* spp. and trypanosomosis. Therefore, a sustainable community‐based tsetse and trypanosomosis control program should be put into place to lessen the impacts of trypanosomosis and *Glossina* activity.

## Graphical abstract

In the Dara district of the Sidama region, cattle production is still seriously threatened by *Glossina* spp. and trypanosomosis. Therefore, a sustainable community‐based tsetse and trypanosomosis control program should be put into place.

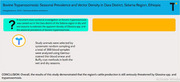

## 1 Introduction

In Ethiopia, a significant portion of agriculture, comprising between 40% and 45% of the overall value of agricultural output, is derived from livestock. Approximately 80% of the value added to livestock comes from cattle. According to the FAO (2019), cattle are an important source of animal protein, energy for households, power for crop production, manure for farmlands, security in the event of crop failure and a way to accumulate wealth. However, endemic diseases like trypanosomosis prevent it from reaching its full potential.

Animal trypanosomosis is a wasting disease that impairs the health and productivity of livestock in sub‐Saharan Africa (Alsan 2015; Giordani et al. 2016; Constable et al. 2017). It significantly impedes both economic growth and the production of cattle (Wilkowsky 2018). Trypanosomosis directly affects the milk and meat productivity of cattle, reduces fertility and increases abortion as well as mortality rates (Alsan 2015; Chanie, Adula, and Bogale 2013; Swallow 2000). The overall consequence is a reduction in herd size and herd composition (Gechere et al. 2012; Taye et al. 2012). The disease's indirect effects are primarily related to power outages during droughts (Tesfaye et al. 2012; Swallow 2000).

A flagellated, unicellular protozoan parasite of the genus *Trypanosoma* is the cause of trypanosomosis. It is mechanically spread by biting flies and cyclically spread by *Glossina* spp. (Constable et al. 2017; Steverding 2008). According to Taylor, Coop, and Wall (2016), trypanosomes are found in the blood and other bodily fluids of vertebrate hosts. The disease causes pyrexia, anaemia, oedema, immunosuppression, and a variety of neurological abnormalities. Affected animals may finally die from the disease (Constable et al. 2017; Eisler et al. 2004). In addition, trypanosomosis in cattle results in considerable financial losses due to illness, mortality, abortion, infertility, decreased milk yield, and treatment expenses (Alsan 2012; Constable et al. 2017).

In Ethiopia, there are three main pathogenic *Trypanosoma* species that infect cattle: *Trpanosoma congolense*, *Trypanosoma vivax* and *Trypanosoma brucei* (Desquesnes et al. 2022; Gebre et al. 2022; Abebe 2005). There have been reports of five different *Glossina* species from around various parts of the country: *Glossina pallidipes*, *Glossina morsitans*, *Glossina fuscipes*, *Glossina tachinoides* and *Glossina longipennis* (Gebre et al. 2022; Abebe 2005). In the southern region of Ethiopia, *G. pallidipes* was the most often reported and abundant species, according to Gebre et al. (2022). Other documented mechanical vectors are *Tabanus*, *Stomoxys* and Haematopota species.

Trypanosomosis and vector endemic locations (such as wild life conservation zones and rivers) that are conducive to vector multiplication delineate the Dara district, resulting in a greater rate of animal mortality and a lower level of drought power and production loss in the region (DDLDO 2020). No research has been done on the prevalence of trypanosomes in cattle or the distribution of vectors in the Dara area, despite studies being done to estimate the prevalence of trypanosomosis and the distribution of vectors in western and south‐western parts of Ethiopia (Leta et al. 2016). Therefore, the aim of the current study was to estimate the seasonal prevalence of cattle trypanosomosis and identify the *Glossina* spp. circulating in the district.

## 2 Materials and Methods

### 2.1 Description of the Study Area

**FIGURE 1:**
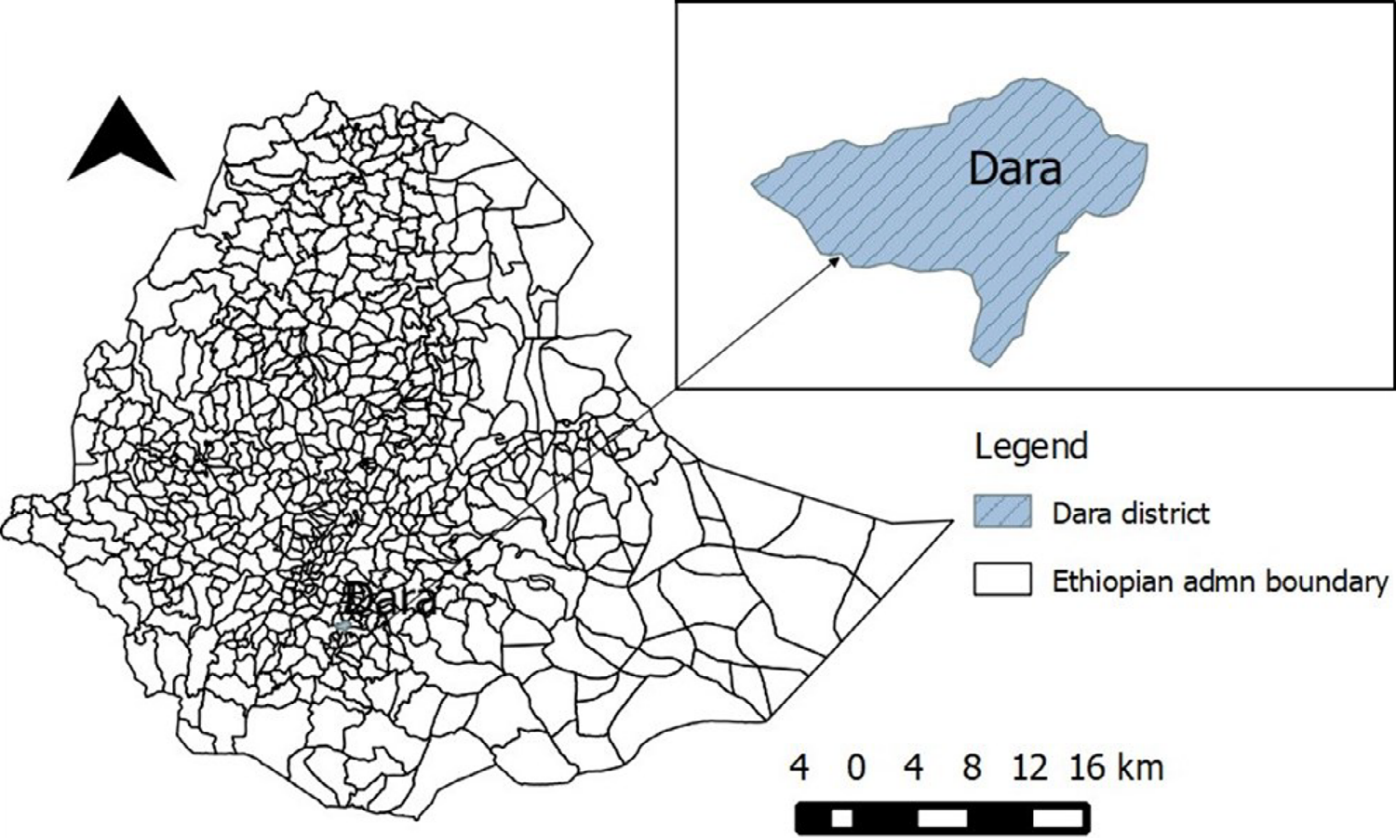
Map showing the study area.

The study was conducted in Dara district of Sidama regional state, southern Ethiopia (Figure 1). The district has good vegetation cover and rivers, which is conducive for *Glossina* spp. habitat. It is located at 6.36°–6.45° N and 38.30°–38.51° E; and the altitude of the district ranges from 900 to 2900 m.a.s.l. The mean annual rainfall is 1201–1600 mm, and the temperature of the area ranges from 12.6°C to 22.5°C. The area is characterised by a bimodal pattern of rainfall: a short rainfall period from April to mid‐June and a long rainfall period from July to mid‐September. The district experiences a distinct dry season that typically starts in October or November, peaks in December, January, and February, and ends in March or April. The area is typically covered by wooded grassland, especially acacia trees and bushes (DDLDO 2020).

Four kebeles (the lowest administrative unit in Ethiopia) were purposefully selected from Dara district, namely Mechisho, Adame, Melinium and Safa, based on their potential for tsetse fly endemicity and complaints of cattle owners.

### 2.2 Study Population

The study population was a local breed of cattle that was kept under an extensive management system and with communal herding. The target population for the study included both sexes and all cattle older than a year. Excluded from the study were animals that had received trypanocidal medication therapy within the previous month and calves (less than a year). The age of the study animals was determined based on the dentition as described by Radostits and Gray (2007) and information from the owners. Three age categories (1–2, 2–5 and > 5 years) were used to group the study cattle.

### 2.3 Study Design, Sample Size and Sampling

A repeated cross‐sectional study design was employed. The study examined several potential risk factors, including age, sex, body condition score (BCS), coat colour and seasons (wet and dry). The Sidama region experiences a distinct dry and wet season pattern. The dry season typically starts in October or November, peaks in December, January and February and ends in March or April. On the other hand, the wet season begins in March or April, peaks in July, August and September with heavy rainfall, and ends in October or November. The first blood sampling and fly capture in this study took place from December to February 2021/2022, and the second sampling was conducted from mid‐May to August 2022. The BCS of the animals was categorised as described by Nicholson and Butterworth (1986) as good, medium and poor. During sample collection, these risk factors were identified and recorded from cattle selected for the study.

The total sample size required for the study was computed by considering the expected prevalence of 14.8% that was reported by Bezabih, Shabula, and Beyene (2015), 95% confidence interval (CI) and 5% absolute precision. The desired sampling size was calculated by using the formula given by Thrusfield (2018).

$$n=\frac{1.96^{2}\times Pexp\left(1-Pexp\right)}{d^{2}}$$

where *n* is required sample size, *P*_exp_ is expected prevalence, *d* is desired absolute precision at 5% and 1.96 is the value of *Z* at 95% CI. Therefore, the calculated sample size was 194 per seasons, and a total of 388 animals were sampled in both wet and dry seasons.

The study animals were selected by systematic random sampling technique as described by Thrusfield (2018). The sampling interval (*K*) is computed as the study population size divided by the required sample size. The first element is chosen randomly among the first *K* elements, and then every *K*th element after that is included in the sample (Dohoo, Martin, and Stryhn 2003). The total cattle population in selected kebeles was 14,562 (i.e., data obtained from the district livestock and fishery development office). So, the first sample was selected randomly, and then every 37th animal (14,562/388 = 37) was selected from the population.

### 2.4 Study Methodology

#### 2.4.1 Parasitological Examination and Packed Cell Volume Measurement

Trypanosomes were detected in blood samples using the buffy coat method following the methods described by Murray et al. (1977). The study animals were restrained, marginal ear veins were cleaned and then punctured with a blood lancet. And blood samples were collected by using heparinised microhaematocrit tubes (i.e., about three‐fourths of the microhaematocrit tubes were filled with the blood), and then after, one side of the tube was sealed with a crista seal (Hawksley Ltd., Lancing, UK). The samples were labelled with all the necessary information. The microhaematocrit with blood was centrifuged for 5 min at 1200 revolutions per minute. It was removed from the haematocrit centrifuge, and the packed cell volume (PCV) was measured using the haematocrit reader. Then, the readings were expressed as a percentage of packed red cells to the total volume of whole blood. Animals with PCV ≤ 24% were considered to be anaemic (Marcotty et al. 2008).

For trypanosome examination, the microhaematocrit tube was cut at about 1 mm below the buffy coat and the contents of the tube were expressed onto a microscopic slide, mixed and covered with a 22 × 22 mm cover slip. Finally, the slide was examined under a 40× objective and 10× eyepiece for movement of parasites (Paris, Murray, and McOdimba 1982; Uilenberg 1998), and trypanosome species were identified based on their movement pattern during the buffy coat examination as described by Murray et al. (1977) and Paris, Murray, and McOdimba (1982). Further smears of the buffy coat were prepared from those animals positive in BCT and giemsa stained for morphological identification of *Trypanosome* spp. (Uilenberg 1998).

#### 2.4.2 Entomological Survey

Eighty NGU traps were placed near trees and/or shrubs in areas used for grazing and watering in preparation for the entomological survey. Three‐day‐old cow urine and acetone were used to evenly bait the traps. The traps were set up at 200–250 m apart and were left in place for 2 days. To prevent tsetse fly damage in the cage, grease was applied to the lowest portion of each trap pole to stop ants from climbing it towards the gathering cage.

Following the sorting, counting and identification of every captured fly, *Glossina* spp. were identified using their morphological traits (Uilenberg 1998; Pollock 1992). Using a hand lens and stereomicroscope, the posterior end of the ventral side of the abdomen was observed to identify female and male *Glossina* spp. The biting flies were identified based on Wall and Shearer's (2001) description. For both *Glossina* species and other biting flies, the apparent *Glossina* density—the quantity of flies caught in each trap each day—was calculated independently (Leak 1999). An apparent density (AD) of the tsetse fly was calculated using the formula: fly catch/trap/day.

### 2.5 Data Management and Analysis

All collected data, parasitological and entomological, were recorded in a Microsoft Excel spread sheet and edited. The data were summarised by descriptive statistics like frequency and mean. STATA version 14.2 computer software was applied for the statistical analysis. The prevalence of trypanosomosis was calculated by dividing the number of infected animals by the total number of examined animals and then multiplied by 100 (Thrusfield 2018). Logistic regression analysis was used to examine the relationship between the risk factors (explanatory variables) and trypanosome infection. Those variables with *p* < 0.25 in the univariable logistic regression analysis were subjected to multivariable logistic regression analyses after the collinearity was checked. Finally, the Hosmer–Lemeshow goodness‐of‐fit test was used to evaluate the model's fitness (Dohoo, Martin, and Stryhn 2003). The receiver operating characteristic curve (ROC) was utilised to assess the fitted model's reliability in more detail. The study took into account 95% CI and 5% desired level of precision. The apparent tsetse density was expressed as the number of flies per trap per day.

## 3 Results

### 3.1 Parasitological Examination

Out of the 388 cattle examined using the buffy coat method in the Dara district, 17 tested positive for trypanosomosis, resulting in an overall apparent prevalence of 4.4% (95% CI = 2.7–6.9). Seasonally, the apparent prevalence was 1.5% in the dry season and 7.2% in the wet season (Table 1).

**TABLE 1:** Overall and seasonal prevalence of trypanosomosis in Dara district, Sidama.

Season	No. examined	No. positive	Prevalence (95% CI)	Std. Err.	OR	*p* value
Dry	194	3	1.5% (0.05–4.7)	—	Ref.	
Wet	194	14	7.2% (4.3–11.8)	0.02	4.95	0.013
Total	388	17	4.4% (2.7–6.9)			

Abbreviations: CI, confidence interval; No, number; OR, odds ratio.

In the research area, two trypanosome species—*T. congolense* and *T. vivax*—were discovered to be in circulation. The prevalence of *T. congolense*, *T. vivax* and mixed infection (*T*. *congolense* and *T*. *vivax*) were 1.6% (95% CI = 0.3–2.8), 1.3% (95% CI = 0.2–2.4) and 1.6% (95% CI = 0.3–2.8), respectively.

### 3.2 Risk Factor Analysis

With univariable logistic regression, the relationship between trypanosome infection and the possible risk factors taken into consideration for this study (i.e., season, sex, age, BCS, and coat colour) was evaluated. In the univariable logistic regression analysis, season, BCS, and coat colour were revealed to be significantly associated (*p* < 0.05) with trypanosome infection in cattle (Table 2).

**TABLE 2:** Univariable and multivariable logistic regression analysis for potential risk factors and trypanosome infection in Dara district.

Risk factors	Category	No. examined	No. positive	Prevalence % (95% CI)	Univariable		Multivariable	
					OR	*p* value	OR	*p* value
Season	Dry	194	3	1.5 (0.5–4.7)	Ref	—	—	—
	Wet	194	14	7.2 (4.3–11.8)	4.95	0.013	5.12	0.013*
Age	≤ 2 years	28	2	7.1 (1.7–25.1)	1.19	0.428	—	—
	2–5 years	134	5	3.7 (1.6–8.7)	Ref	—	—	—
	> 5 years	226	10	4.4 (2.4–8.1)	1.98	0.751	—	—
Sex	Male	44	2	4.5 (1.1–16.7)	1.04	0.955	—	—
	Female	344	15	4.4 (2.6–7.1)	Ref	—	—	—
Coat colour	White/Spotty	96	2	2.1 (0.5–8.0)	0.29	0.774	0.67	0.750
	Gray	68	1	1.5 (0.2–9.9)	Ref	—	—	—
	Black	92	11	12.0 (6.7–20.4)	2.37	0.018	6.81	0.016*
	Red/brown	132	3	2.3 (0.7–6.9)	0.10	0.923	1.21	0.836
BCS	Good	128	2	1.6 (0.4–5.1)	Ref	—	—	—
	Medium	157	5	3.2 (1.3–7.5)	2.07	0.389	2.27	0.351
	Poor	103	10	9.7 (5.2–17.2)	6.77	0.015	7.45	0.015*

Abbreviations: BCS, body condition score; CI, confidence interval; No., number; OR, odds ratio.

All of the explanatory variables for trypanosome infection are non‐collinear, according to a multicollinearity test. Season, coat colour, and BCS were, therefore, the explanatory variables with *p* > 0.25 that were chosen for the multivariable logistic regression model. The final model revealed that a Hosmer–Lemshow χ^2^ = 2.32, *p* = 0.9697 and ROC = 8223. As a result, cattle were 5.12 times more likely to be infected in the wet than a dry season; cattle with black coats were 6.81 times more likely to be infected with trypanosomes than those with other coats; and poor‐body‐conditioned cattle were 7.45 times more likely to be infected with trypanosome infection.

### 3.3 Haematological Examination Result

The overall mean PCV of studied cattle was 27.4% (95% CI = 27.0–27.9); and the mean PCV of infected animals was 21.2%. According to the analysis of PCV values in the animals tested for trypanosome infection, the mean PCV value for the parasitemic cattle was 21.2 ± 0.5 SE (95% CI = 20.2–22.3), whereas the mean PCV value for the aparasitemic cattle was 27.7 ± 0.2 SE (95% CI = 27.2–28.2) (Table 3). There were 88 (22.68%) animals with PCV < 24% and 300 (77.32%) cattle with PCV > 24% (non‐anaemic).

**TABLE 3:** Mean PCV value for parasitaemic and aparasitaemic cattle.

Status	No. examined	Mean PCV (95% CI)	Std. Err.	OR	*p* value
Aparasitaemic	371	27.7% (27.2–28.2)	0.24	66.4	0.000
Parasitaemic	17	21.2% (20.2–22.3)	0.50	Ref	
Overall	388	27.4% (27.0–27.9)	0.24		

Abbreviations: CI, confidence interval; No., number; OR, odds ratio; PCV, packed cell volume.

### 3.4 Entomological Survey

**FIGURE 2:**
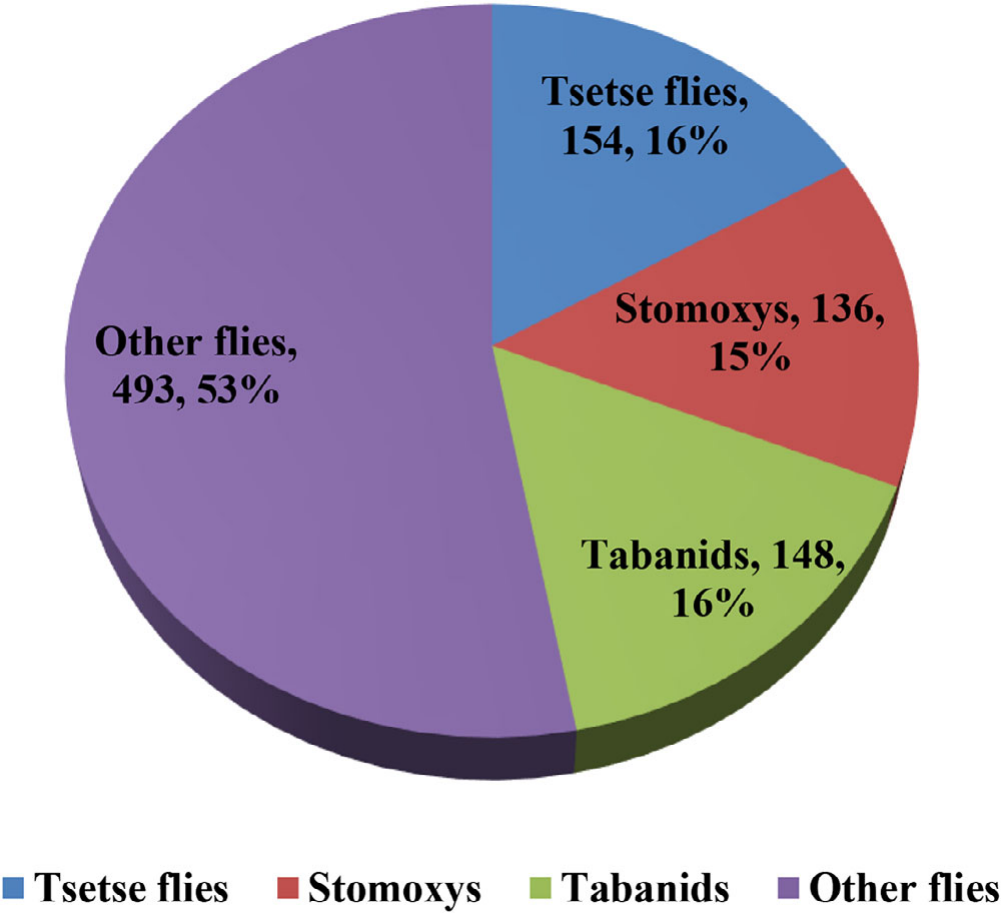
Number and proportion of tsetse and other flies captured in entomological survey.

In the study, a total of 931 flies were captured during both dry and wet seasons using 80 NGU traps over 2 days. Of these, 154 (16%) were tsetse flies belonging to *G. pallidipes*, 148 (16%) were *Tabanids*, 136 (15%) were *Stomoxys*, and 493 (53%) were other biting flies (Figure 2). The overall AD of the tsetse flies was 0.96 F/T/D, while the AD of *Tabanids* was 0.93 F/T/D and of *Stomoxys* spp. was 0.85 F/T/D.

## 4 Discussion

During the course of two seasons, this study explored bovine trypanosomosis and vector density in the Dara district, revealing moderate trypanosomosis prevalence and tsetse fly abundance. The results of the current investigation demonstrated that cattle production is threatened by *Glossina* spp. and trypanosomosis in the study area. Compared to earlier research conducted in the country using conventional parasitological diagnostic methods, the study prevalence (4.4%) is consistent with 5.1% by Sheferaw et al. (2019) and 4.3% by Efrem et al. (2023). The current result, however, is lower than the 8.2% prevalence reported in a previous study conducted in the same region (Abebe, Dessie, and Solomon 2021). The 4.4% prevalence is also lower than the prevalence of other studies in the country, such as 7.2% by Sheferaw et al. (2016) and 7.8% by Abebe, Gute, and Simon (2017). Moreover, the current study prevalence falls behind the pooled prevalence (8.12%) from a meta‐analysis of bovine trypanosomosis studies conducted from 1997 to 2014 (Leta et al. 2016). The variation in prevalence among studies may be due to factors such as differences in tsetse density, sampling season, ecological conditions of study regions, trypanocidal drug usage, and fly control practices.

The study prevalence was considerably greater during the wet season. According to multiple investigators (Seyoum et al. 2022; Eyasu, Mekuria, and Sheferaw 2021; Mekuria et al. 2021; Okello et al. 2022; Majekodunmi et al. 2013), there is a higher prevalence during the wet season. The higher prevalence of trypanosomosis during the wet season is associated with the increased number of *Glossina* spp. and other biting flies captured during this period (Gachoki et al. 2023). The number of tsetse and other biting flies tends to increase during the wet season. A rise in the vector population may also advance the Trypanosoma prevalence by increasing the rate of transmission.

It has been found that cattle with black coats are more likely to be affected by trypanosomosis because *Glossina* spp. prefer to land on black objects more often than any other colour (Seifert 1996). This finding is consistent with previous studies conducted in Ethiopia (Sheferaw et al. 2016; Abebe, Gute, and Simon 2017; Mekuria et al. 2021; Seyoum et al. 2022).

Although chronic infection with trypanosome is characterised by a poor body condition score (Constable et al. 2017), the acute form could occur in cattle with a good body condition score (Joan et al. 2020). This research highlights that the average PCV value was significantly lower in animals infected with trypanosomes in animals sampled during the dry season and in poorly conditioned animals than their counterparts. The detrimental impact of trypanosome infection, limited availability of food in the dry season, and increased exposure to trypanosomes due to higher tsetse fly populations likely contribute to these reduced PCV values. These results are consistent with findings from other studies in various regions (Marcotty et al. 2008; Sheferaw et al. 2016; Abebe, Gute, and Simon 2017; Mekuria et al. 2021; Seyoum et al. 2022; Efrem et al. 2023)

The study revealed that *T. congolense* and *T. vivax* were responsible for bovine trypanosome infection in the Dara district. These two species of *Trypanosoma* were commonly reported from the southern and western parts of Ethiopia (Gebre et al. 2022; Eyasu, Mekuria, and Sheferaw 2021; Mekuria et al. 2021; Degneh et al. 2017; Leta et al. 2016; Sheferaw et al. 2016; Eshetu, Barata, and Butako 2017; Rundassa, Menkir, and Kebede 2013; Gechere et al. 2012).

*G. pallidipes* was the only tsetse species and the predominant vector identified in this research during both the dry and wet seasons. Various reports revealed that *G. pallidipes* is widespread and endemic in the southern part of the country (Gebre et al. 2022; Seyoum et al. 2022; Sheferaw et al. 2019; Gechere et al. 2012; Zeleke 2011) and in East Africa. The sole detection of *G. pallidipes* is probably associated with the woody grassland vegetation characteristic of the area, providing suitable breeding and foraging grounds for the flies. These shaded habitats offer resting spots for *G. pallidipes* and opportunities to target host species in open areas (Gachoki et al. 2023). Consistent with our findings, a previous study in the same area (Abebe, Dessie, and Solomon 2021) and other studies in similar agro‐ecological regions nearby similarly established *G. pallidipes* as the sole tsetse type (Seyoum et al. 2022).

The higher density of other biting flies than *G. pallidipes* signifies the possibility of mechanical transmission of trypanosomosis caused by *T. vivax* in the study areas, even in the absence of *Glossina* species in cattle and other domestic animals in tsetse‐free regions, which is well understood (Desquesnes and Dia 2004; Cherenet et al. 2004). Thus, the identification of these potential vectors emphasises the complex and varied nature of the transmission dynamics of animal trypanosomosis in the area.

## 5 Conclusion and Recommendations

The study reported a bovine trypanosomosis prevalence of 4.4%, which remains a significant issue that affects cattle productivity and livelihood in the Sidama region of the Dara area. According to the study, *T. congolense* and *T. vivax* were the main causes of bovine trypanosomosis in the studied location, and *G. pallidipes* was the primary cyclical vector of transmission. The presence of biting flies could have contributed to the epidemiology of bovine trypanosomosis, especially during the dry season. The decreased PCV values observed among trypanosome‐infected cattle indicate that the parasite could affect the general well‐being of the animals, including severe anaemia. Therefore, it is necessary to strengthen the current vector and parasite control efforts in the study area and to take mechanical vectors into account in the strategy.

## Author Contributions

**Tafese Jiso**: conceptualization, investigation, data curation, formal analysis, visualization, writing–original draft, methodology, software. **Gizachew Hailegebreal**: validation, supervision, writing–review and editing, conceptualization, methodology, investigation. **Dessie Shiferaw**: supervision, validation, writing–review and editing, methodology, investigation, conceptualization.

## Ethics Statement

The animal studies were approved by Hawassa University College of Natural and Computational Sciences Research Ethics Review Committee with approval number HU/CNCS/ERC 113/2021. The studies were conducted in accordance with the local legislation and institutional requirements. Written informed consent was obtained from the owners for the participation of their animals in this study.

## Conflicts of Interest

The authors declare no conflicts of interest.

### Peer Review

The peer review history for this article is available at https://publons.com/publon/10.1002/vms3.70130.

## Data Availability

The original contributions presented in the study are included in the article/supplementary material; further inquiries can be directed to the corresponding author/s.
